# Supplementary material for: GWAS supported by computer vision identifies large numbers of candidate regulators of in planta regeneration in Populus trichocarpa
Source: G3 (Bethesda). 2024 Feb 7;14(4):jkae026. doi: 10.1093/g3journal/jkae026 (PMC10989874; doi:10.1093/g3journal/jkae026)

**Supplementary Figure 14. Genome browser views of ART associations.** Association mapping with ART was followed by inspection of peaks and their alignment with gene annotations to find cases where ART peaks implicate specific genes. Nine examples are shown.


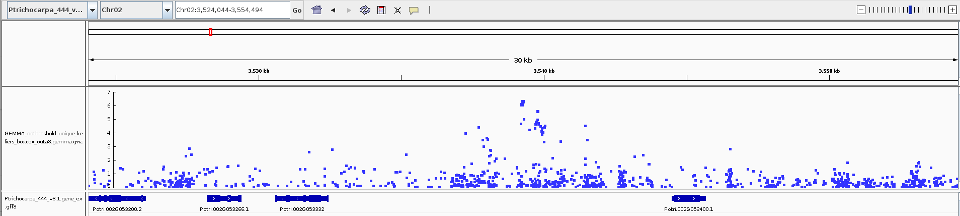


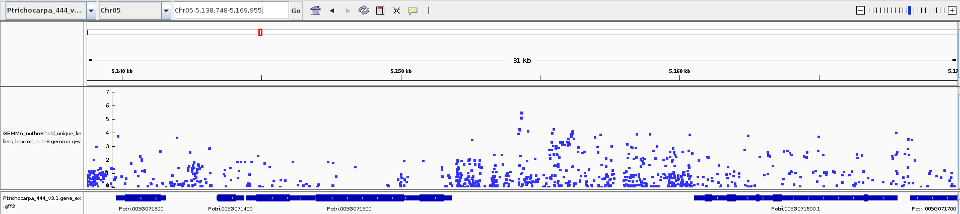


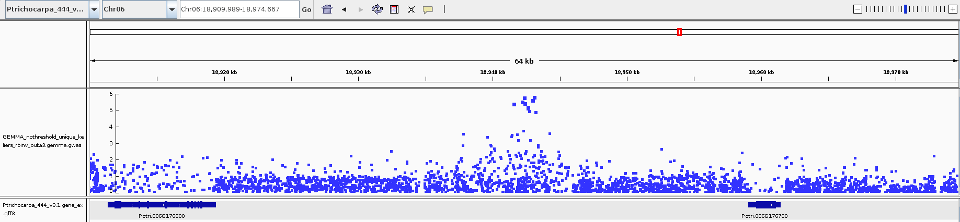


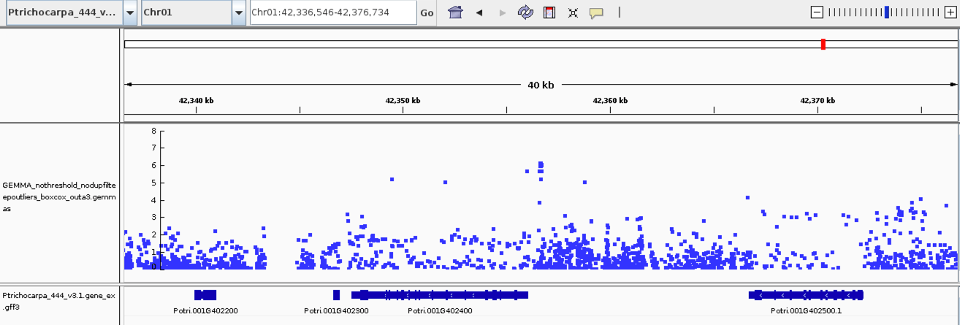


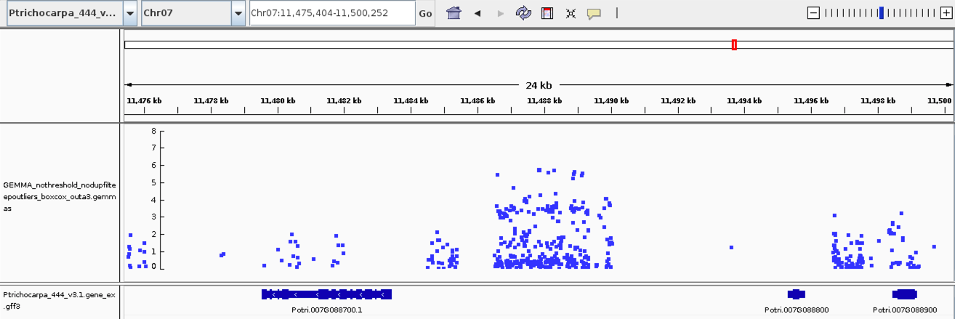


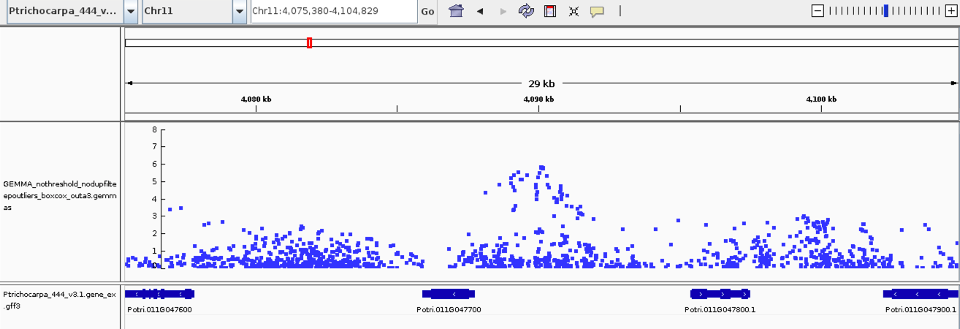


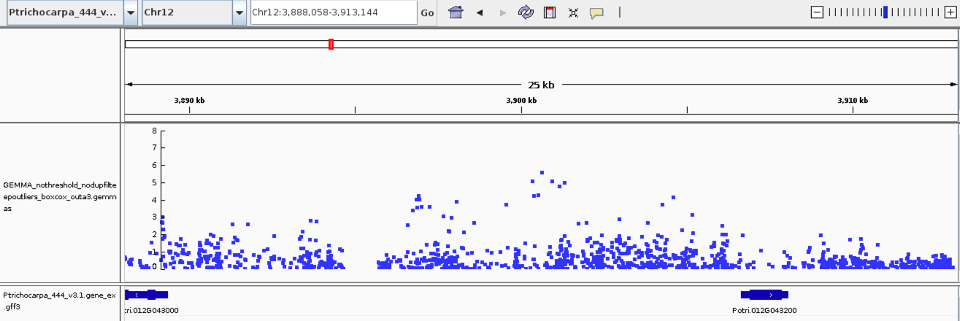


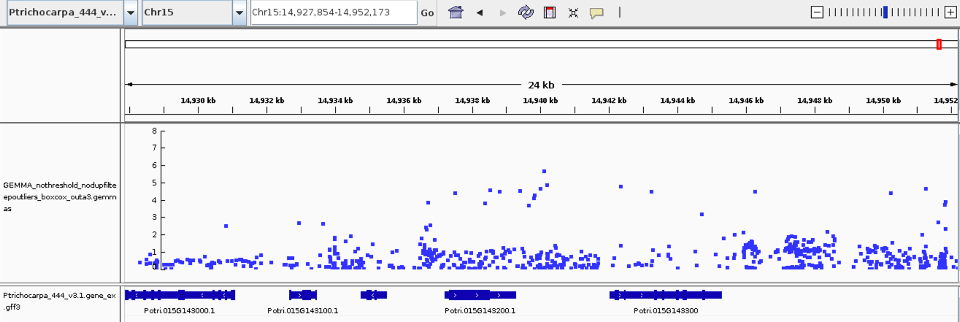


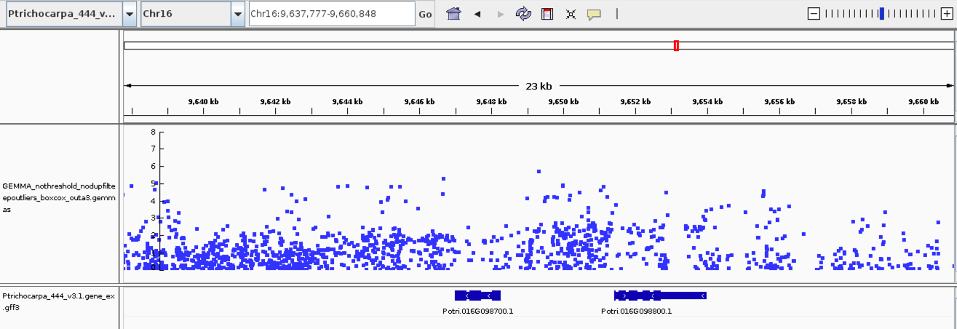

Supplement: jkae026_Supplementary_Data [file jkae026_supplementary_data.zip › Figure_S14_G3-2023-404699.docx]
