# Supplementary material for: GWAS supported by computer vision identifies large numbers of candidate regulators of in planta regeneration in Populus trichocarpa
Source: G3 (Bethesda). 2024 Feb 7;14(4):jkae026. doi: 10.1093/g3journal/jkae026 (PMC10989874; doi:10.1093/g3journal/jkae026)
Supplement: jkae026_Supplementary_Data [file jkae026_supplementary_data.zip › File_S1_G3-2023-404699.docx]

### Supplementary methods and materials

#### Association Mapping

Prior to GEMMA, SNPs were filtered based on minor allele frequency (MAF) > 0.01 and a missing rate of given SNPs across genotypes < 0.10 using PLINK (Purcell *et al.*, 2007), resulting in ~13.2 million SNPs. Filtering criteria were based on the whole SNP set of 1323 genotypes. GEMMA was used to compute Wald *p*-values for SNP effects, using the `-lmm 1` option. The number of SNPs analyzed by GEMMA was further reduced to ~12.5 million because GEMMA performed additional filtering with a default r^2^ threshold of 0.9999 and MAF and missing rates of 0.01 and 0.10, which were recomputed at this stage. In addition to performing association mapping, GEMMA was used to provide an estimate of narrow-sense SNP heritability (*h^2^_SNP_*) for each trait. Downstream GWAS and gene candidate evaluation was performed for traits with estimated *h^2^_SNP_* above 0.10.

Second, we utilized logistic models for dichotomized traits with Generalized Mixed Model Association Test (GMMAT; (Chen *et al.*, 2016). Due to the computational expense of computing Wald *p*-values via logistic regression, we first performed the GMMAT variance component score test (`glmm.score`) for a genome-wide screen and then extracted a subset of 100 or 1,000 SNPs with the lowest score test *p*-values from each run and computed Wald *p*-values for these (using `glmm.wald`). This GMMAT workflow was performed with two SNP subsets prepared by PLINK: one had a missing rate threshold of 0.10 and an MAF threshold of 0.05 (~7.7 million SNPs), and the second had the same missing rate threshold but an MAF threshold of 0.01 (~13.2 million SNPs). Since rare SNPs led to inflated score tests and interfered with the ability to robustly select SNPs for downstream Wald tests, the former SNP set was relied upon to study six traits: wk. 2 callus area, wk. 3 callus area, wk. 2 shoot area, shoot PC1, shoot PC2 and callus/shoot PC1.

Finally, for multiple-marker tests, we applied the SNP-set (Sequence) Kernel Association Test (SKAT; Ionita-Laza *et al.*, 2013) with untransformed traits. SKAT was performed on overlapping 3kb windows staggered by 1kb, using a set of 34.0 M SNPs filtered for a missing rate of 15%. The R extension Multi-Threaded Monte Carlo SKAT (MTMCSKAT) was used to run SKAT on a high-performance cluster, COMET (made available through NSF XSEDE (Towns *et al.*, 2014). We calculated empirical *p*-values for top associations to avoid Type I and Type II error resulting from the non-normal distributions of untransformed traits. Two means of controlling for population structure were tested and compared with this workflow. We compared a “P” model in which structure is represented by principal components derived from SNPs (computed with PLINK) to a “Q” model in which structure is alternatively represented by subpopulation estimates produced by fastSTRUCTURE (Raj *et al.*, 2014).

To produce PCs for the P model, we employed a filtered set of ~10.3M SNPs with MAF > 0.05 and consulted scree plots and used K-means clustering to inform about the number of PCs appropriate for representing population structure; as a result we used 6 PCs for the P model.

To produce a Q matrix for use with SKAT Q models, we used fastSTRUCTURE using a subset of ~73k SNPs filtered based on LD, MAF, and missing rate using PLINK with parameters ` --indep-pairwise 100kb 10 0.05 --maf 0.05 --geno 0.1`. Ten replicates were performed with fastSTRUCTURE for each possible number of subpopulations (K) ranging from 3 to 12. To understand subpopulations in an evolutionary context, we used SNPhylo (Lee *et al.*, 2014) to produce a dendrogram from our SNP data. SNPhylo was run with a subset of ~123k SNPs prepared by PLINK with parameters ` --indep-pairwise 10kb 10 0.05 --maf 0.05 --geno 0.1`.

Geographical locations (longitude and latitude) were recorded for 1,301 of 1,323 genotypes in the SNP set and plotted against traits, SNP-derived PCs (for SKAT “P” model), primary subpopulation information (for SKAT “Q” model), and dendrogram information (from SNPhylo) using the `phylo.to.map` function in Phytools (R) and Google Maps “My Maps”. Phytools was also used to cross-reference dendrograms with traits, SNP-derived PCs and primary subpopulation information (using function `phylo.heatmap`; Revell, 2012).

To inform about the appropriate window size for SKAT, as well as to inform about the likelihood of genes proximal to associated SNPs or SNP windows being directly involved in affecting traits (vs. being associated as a result of genetic linkage), we evaluated LD decay. To facilitate efficient computation of LD decay, a reduced SNP set (~78k SNPs) was prepared by PLINK with parameters `--maf 0.05 –geno 0.1 –thin 0.01`. Further reduced SNP files were prepared with PLINK to only include genotypes in the “Oregon” and “California” subpopulations (named based on general location of most genotypes in each). PLINK was used to calculate pairwise LD between SNPs on the same chromosome for each of these three filtered SNP sets. Using R, the average LD for each possible distance (e.g. 1bp, 2bp, 3bp… up to 50kb) was computed and plotted for the whole population as well as each of the two selected subpopulations.

#### Evaluation of relationships between regeneration traits, subpopulations and geography

Following the identification of subpopulation structure when fastSTRUCTURE was used to produce covariates for the SKAT “Q” model, we aimed to further investigate the relationships between traits, geography and theoretical ancestral subpopulations to gain insights into the possible adaptive evolution of these regeneration traits. To this end, we used `lm` in R to construct linear models regressing each trait over latitude and the Q matrix featuring estimates of each theoretical ancestral subpopulation’s contribution to each individual’s genome (from fastSTRUCTURE). We then visualized relationships, latitude and subpopulation using `ggplot2` in R.

### Supplementary results and discussion

#### Distinct ancestral subpopulations supported by population structure and phylogeography analysis

Relationships between evolutionary clades, geography, and population structure suggest that *P. trichocarpa*, despite its dioecy and long-distance gene flow, exists with a number of subpopulations that are statistically distinct albeit highly admixed. A total of 120 fastSTRUCTURE runs were performed, including 10 replicates for each value of K (subpopulation number) ranging from 2-13. The log marginal likelihood appears to be maximized with K equal to 6 or 7 (Fig. S8). For each individual in the population, the most closely related subpopulation was extracted and considered the primary subpopulation. Geographic and evolutionary patterns were revealed by cross-referencing of a dendrogram (SNPhylo; Lee *et al.*, 2014) with primary subpopulation and geographic location. These plots were evaluated with primary subpopulations from fastSTRUCTURE models both with K=6 and K=7 (Fig. S9); the K=7 model showed the strongest alignment between phylogeny and geography. Approximately from Seattle northward, individuals display a heavy degree of admixture and fail to cluster into clear subpopulations. Otherwise, the existence of several subpopulations is supported by agreement between phylogenetic clades, geographic location, and primary subpopulation label from fastSTRUCTURE. These include distinct subpopulations in the western region of Idaho and nearby eastern Oregon and Washington (and extending all the way to the eastern Washington Cascades near Yakima), the Willamette Valley of central western Oregon and nearby Western Washington, southwest Oregon and nearby northern California, northwestern Washington extending into southwestern Canada, and central western to northwestern Canada (Fig. S10). Also of note, we found evidence that LD rates vary across groups of distinct theoretical ancestral subpopulations, as shown by LD curves fit for “Oregon” and “California” groups (named by approximate location of primary theoretical ancestral subpopulation; Fig. S7). A linear model fit over this data for both groups, with an interaction term between primary ancestral subpopulation and a spline function of LD decay, indicated that this difference was statistically significant (empirical *p*-value < 0.001, 1000 permutations).

We further attempted to summarize population structure by performing PCA over SNP data using PLINK. Similar to fastSTRUCTURE subpopulation estimates, PCs explaining a substantial portion of variance show clear relationships with geography and most of the same phylogenetic clades (Fig. S11). The use of six PCs to represent population structure in SKAT models, as discussed below, was supported by the scree plot (Fig. S3) and the relatively minor contributions of subsequent PCs to k-means clusters computed from PCs (Fig. S4).

We attempted to gain insights into the possible role of evolution in regeneration traits via relationships between the traits, latitude and theoretical ancestral subpopulation (Methods). At α = 0.005, there appears to be a significant effect of latitude of clone origin on the trait of callus area at week four, while controlling for subpopulation. Several other relationships are significant at 0.05, between various callus traits and latitude and/or subpopulation (Table S3). Visualization of the relationships between traits and latitude along with regression trendlines showed a positive relationship between many regeneration traits and increasing latitude (Fig. S13). Considering the lack of independence between variables of theoretical ancestral subpopulation and latitude, we advise caution in overinterpreting these results as evidence of either genetic drift or an adaptive role of regeneration, but also note several significant or borderline-significant trends indicating such a role may exist. We speculate that possible contributions of geography to evolutionary advantages for regeneration may relate to tissue damage resulting from freezing or predators.

#### Distinct subpopulations correlate with phylogeography

The existence of distinct ancestral subpopulations of *P. trichocarpa* and a relationship of these subpopulations with geography is supported by cross-referencing of results from population structure analysis (fastSTRUCTURE), phylogenetics (SNPhylo), and geographical information for genotypes. These distinct subpopulations appear clearly in the southern portion of the population, whereas the northern portion displays a remarkable degree of admixture with mixed origins across the southern subpopulations. We speculate that, following the establishment of distinct southern subpopulations during the Last Glacial Period (Armstrong *et al.*, 1965), the recession of glaciers allowed for these subpopulations to spread to the northern region—where there has not yet been sufficient time or subdivision for distinctive populations to form. In contrast, the disjunct nature of many of the southern population groups is likely to have provided historical opportunities for differentiation. While previous work using approximately 12 isozyme loci did not reveal distinct subpopulations of *P. trichocarpa* over a more narrow, but similar geographical range (Weber & Stettler, 1981), our work demonstrates the much-increased power of genome-scale SNP data—where millions of loci are surveyed—to detect subpopulations.

#### Similar results from SKAT with either PC or fastSTRUCTURE covariates

We compared results from complementary SKAT models with population structure represented either by the fastSTRUCTURE Q matrix with 7 subpopulations (“Q model”) or by the first 6 PCs (“P model”) for a subset of four traits (callus area at wk. 4 and wk. 5; shoot area at wk. 4 and wk. 5). These models displayed a remarkable level of agreement, especially for *p*-values that met thresholds of significance and were thus selected for validation by computing empirical *p*-values with MTMCSKAT (Fig. S5).

#### Overlap with genes implicated from published GWAS analyses of regeneration

The candidates we identified showed very little similarity to results from related work. In prior work, GWAS was performed in 280 genotypes of *P. trichocarpa* to study traits related to in vitro callus regeneration. This study yielded eight candidate genes, none of which appear among our results (Tuskan *et al.*, 2018). A GWAS of traits related to roots and vegetative shoots in *Populus deltoides × simonii* with 434 genotypes produced 233 QTLs and multiple candidate genes were considered within proximity of each QTL, yielding a total of 595 unique candidate genes, only three of which were also found among traits analyzed in our study. Potri.015G018200, encoding a putative protein kinase, is a gene candidate from our analysis of callus area at week two as well as a prior analysis of a measurement of the number of leaves per vegetative shoot in *P. euphratica*. This leaf number trait also yields an association for Potri.004G156900, encoding a putative RETICULATA-related protein also appearing as a candidate in our analysis of shoot area at week four. Another association is with Potri.019G035200, which encodes an oxygenase involved in heme degradation within chloroplasts; it was found among our candidate genes for callus at week two as well as in the same prior work for average stem diameter (Sun *et al.*, 2019).

In a review of GWAS of regeneration in diverse species, Lardon and Geelen (Lardon & Geelen, 2020) noted that candidate genes identified across studies are non-overlapping to a great extent. Some of the potential causes for the low degree of overlap include genetic differences between study populations, variation in tissue or explant physiology, variation in the treatments used to promote regeneration, random variation in detection given underpowered statistics and numerous genes under polygenic control, and differing statistical approaches (Lardon & Geelen, 2020). All of these factors would apply to our study vs. the other published work in *Populus*. Another likely contributor to lack of overlap is that our GWAS is the only one studying *in planta* regeneration, as opposed to *in vitro* regeneration or vegetative shoot development, and the genetic control of these developmental processes is likely to vary significantly. Finally, we note that the traits obtained from our computer vision pipeline are distinct from those in these prior studies, which made use of various manual scoring systems.

**References cited in supplementary text**

**Armstrong JE, Crandell DR, Easterbrook DJ, Noble JB**. **1965**. Late Pleistocene Stratigraphy and Chronology in Southwestern British Columbia and Northwestern Washington. *GSA Bulletin* **76**: 321–330.

**Chen H, Wang C, Conomos MP, Stilp AM, Li Z, Sofer T, Szpiro AA, Chen W, Brehm JM, Celedón JC, *et al.*** **2016**. Control for Population Structure and Relatedness for Binary Traits in Genetic Association Studies via Logistic Mixed Models. *The American Journal of Human Genetics* **98**: 653–666.

**Ionita-Laza I, Lee S, Makarov V, Buxbaum JD, Lin X**. **2013**. Sequence Kernel Association Tests for the Combined Effect of Rare and Common Variants. *American Journal of Human Genetics* **92**: 841–853.

**Lardon R, Geelen D**. **2020**. Natural Variation in Plant Pluripotency and Regeneration. *Plants* **9**: 1261.

**Lee T-H, Guo H, Wang X, Kim C, Paterson AH**. **2014**. SNPhylo: a pipeline to construct a phylogenetic tree from huge SNP data. *BMC Genomics* **15**: 162.

**Liu X, Huang M, Fan B, Buckler ES, Zhang Z**. **2016**. Iterative Usage of Fixed and Random Effect Models for Powerful and Efficient Genome-Wide Association Studies. *PLOS Genetics* **12**: e1005767.

**Purcell S, Neale B, Todd-Brown K, Thomas L, Ferreira MAR, Bender D, Maller J, Sklar P, de Bakker PIW, Daly MJ, *et al.*** **2007**. PLINK: A Tool Set for Whole-Genome Association and Population-Based Linkage Analyses. *The American Journal of Human Genetics* **81**: 559–575.

**Raj A, Stephens M, Pritchard JK**. **2014**. fastSTRUCTURE: variational inference of population structure in large SNP data sets. *Genetics* **197**: 573–589.

**Revell LJ**. **2012**. phytools: an R package for phylogenetic comparative biology (and other things). *Methods in Ecology and Evolution* **3**: 217–223.

**Sun P, Jia H, Zhang Y, Li J, Lu M, Hu J**. **2019**. Deciphering Genetic Architecture of Adventitious Root and Related Shoot Traits in Populus Using QTL Mapping and RNA-Seq Data. *International Journal of Molecular Sciences* **20**: 6114.

**Towns J, Cockerill T, Dahan M, Foster I, Gaither K, Grimshaw A, Hazlewood V, Lathrop S, Lifka D, Peterson GD, *et al.*** **2014**. XSEDE: Accelerating Scientific Discovery. *Computing in Science & Engineering* **16**: 62–74.

**Tuskan GA, Mewalal R, Gunter LE, Palla KJ, Carter K, Jacobson DA, Jones PC, Garcia BJ, Weighill DA, Hyatt PD, *et al.*** **2018**. Defining the genetic components of callus formation: A GWAS approach. *PLoS ONE* **13**.

**Weber JC, Stettler RF**. **1981**. Isoenzyme variation among ten populations of Populus trichocarpa Torr. et Gray in the Pacific Northwest. *Silvae Genetica* **30**: 82–87.
