# Supplementary material for: GWAS supported by computer vision identifies large numbers of candidate regulators of in planta regeneration in Populus trichocarpa
Source: G3 (Bethesda). 2024 Feb 7;14(4):jkae026. doi: 10.1093/g3journal/jkae026 (PMC10989874; doi:10.1093/g3journal/jkae026)
Supplement: jkae026_Supplementary_Data [file jkae026_supplementary_data.zip › Supplemental_table_and_figure_legends_G3-2023-404699.docx]

**Supplementary Figures**

**Figure S1. SNP density plot.** The density of SNPs is shown for a SNP set of approximately 10.3 million SNPs, filtered on the basis of MAF (MAF > 0.05), and limited to SNPs on assembled contiguous chromosomes. This plot is shown with a bin size of 10kb and was generated using `CMplot` (R).

**Figure S2. Trait before and after Box-Cox transformation, given for the example of Callus Area at week 2.** Zero values were excluded before (above) and after (below) transformation. The QQ plots (left) show quantiles of the distribution plotted against theoretical quantiles of normal distributions with the same mean and standard deviation. These theoretical normal distributions are also represented by a black line in the histograms (right).

**Figure S3. Scree plot from PCA over SNP data.**The scree plot is shown with eigenvalues for each of 20 PCs obtained by PCA over 10.3 M SNPs with MAF > 0.05: PCA was performed using PLINK and the plot was produced with `ggplot2` (R).

**Figure S4. Correlations between PCs from PCA over SNP data.** Correlations are shown between top eight PCs from PCA over 10.3M SNPs with MAF > 0.05. K-means clusters (with k = 6) were computed using R and used to group and label samples, with each cluster shown by a different color. This plot was produced with `ggpairs` (R).

**Figure S5. Comparison of P and Q models with SKAT.** Q-Q plots and histograms of p-values are shown, along with comparisons of p-values between the P and Q models and between empirical p-values and p-values before resampling for each method.

**Figure S6. Results from PCA over in planta regeneration traits.** Results are shown for two PCA batches: over all callus traits (A) and shoot traits (B) Heat maps of loadings from PCA (left) show contributions of each trait to each PC. Scree plots (right) show most variation is explained by these top two principal components.

**Figure S7. Linkage disequilibrium decay curves.** Graphs are shown for Oregon (“OR”) subpopulation (group 5 in Fig. S10) and California (“CA”) subpopulation (group 2 in Fig. S10). Primary subpopulations were determined using fastSTRUCTURE, with a K = 7 model (File S1, Supplementary Methods). Each point represents the mean R2 between SNPs of a given distance (x-axis). Lines represent LD decay as computed with a spline function. The difference between the CA and OR rates of LD decay was statistically significant (P < 0.001) based on 1000 permutations of a spline model (File S1, Supplementary Results and Discussion).

**Figure S8. Analysis of subpopulations.** fastSTRUCTURE was performed with 10 replicates, indicating that population structure is best represented with six or seven subpopulations.

**Figure S9. Information on theoretical ancestral subpopulations via fastSTRUCTURE.** (A) K = 7 model and (B) K = 6 model. Subpopulations are cross-referenced with both dendrogram and geography, with primary clones colored by primary subpopulation, for 130 randomly selected clones for readability. This plot was produced using the `phytools` R package. For improved readability, dotted demarcations were manually added to indicate approximate groupings based on fastSTRUCTURE clusters and/or phylogenetic groupings.

**Figure S10. Phylogenetic relationships of natural source populations**. Shown are the theoretical ancestral subpopulations (fastSTRUCTURE, with K = 7 model), cross-referenced with geographical locations of the study clones taken from the wild. Data is shown for the 1,301 clones for which location data is available (out of 1,323). Points are labeled by the theoretical subpopulation accounting for the largest portion of ancestry for each clone. The plot was produced by Google Maps (MyMaps).

**Figure S11. Dendrogram produced by SNPhylo.** Results are cross-referenced with (A) top six principal components from PCA over SNP data and (B) subpopulations from fastSTRUCTURE (K = 7 model). Data is shown for only 130 randomly selected clones for readability.

**Figure S12. Principal component analysis results cross-referenced with geographical locations of clones.** Data is shown for the 1,301 clones for which location data was available (out of 1,323). This plot was produced with Google Maps MyMaps.

**Figure S13. Relationships between callus/shoot area traits and latitude of clone of origin. Clones are labeled by primary subpopulation (fastSTRUCTURE).**A trendline with a 95% confidence interval is displayed for the general relationship between each trait and latitude. These plots were produced using `ggplot2` (R).

**Supplementary Tables**

**Table S1. Effects of transformations on statistical distributions of *in planta* regeneration traits obtained directly by computer vision.** Raw traits were obtained by parsing machine vision outputs into vectors with values of continuous trait for each genotype; all traits were transformed first by excluding zero values, and finally by a Box-Cox transformation. CC = correlation coefficient. Red tones indicate weaker (lower) CC values within rows.

**Table S2. Effects of transformations on statistical distributions of *in planta* regeneration principal component traits obtained by PCA over computer vision traits.** Principal component traits were obtained by performing principal component analysis (PCA) to reduce large numbers of raw traits into a smaller number of variables. These traits were processed by a range of transformations, labeled in the “Transf. type” column. Transformation “A” simply involved excluding genotypes which have a zero value for all raw traits, followed by a Box-Cox transformation. Transformation “B” additionally featured removal of outliers prior to Box-Cox, while “C” involved thresholding at an inflection point (elbow) prior to Box-Cox. CC = correlation coefficient. Red tones indicate weaker (lower) CC values within rows.

**Table S3. Relationships between *in planta* regeneration traits, geography and theoretical ancestral subpopulation.** Shown are *p-*values obtained by regression of Box-Cox-transformed traits over Q matrix (fastSTRUCTURE with K = 7 model) and latitude of origin for each clone. Red tones indicate more significant (lower) *p*–values. A single *p*-value is listed for the intercept and effects for each of seven subpopulations because the *p-*values for the intercept and subpopulation effects were the same for each given trait. The directions of relationships between each trait and latitude can be seen in Fig. S13.

**Table S4. Narrow-sense SNP heritability of *in planta* regeneration traits as computed by GEMMA.** Traits are listed with their and corresponding heritabilities and transformations. Several traits were analyzed with multiple transformations and/or pre-treatments due to a lack in clarity on which transformations were optimal.

**Table S5. Tallies and statistics of QTL peaks found significant across methods, traits and significance thresholds.** Summaries of genes encompassing or near QTLs are shown for each combination of GWAS method and trait yielding associations that are significant according to a given criteria (conservative Bonferroni, FDR = 0.10, or ART-Bonferroni). For SKAT and ART associations, the distance shown is that from SNP window centers to the nearest genes. Otherwise, the distance is that between given SNPs and genes.

**Table S6. *In planta* callus and shoot regeneration associations passing Bonferroni and/or FDR (0.10) thresholds.**

**Table S7. *In planta* callus and shoot regeneration associations passing ART-Bonferroni threshold (GEMMA).**

**Table S8. *In planta* callus and shoot regeneration associations passing ART-Bonferroni threshold (GMMAT).**

**Table S9. Abbreviations and acronyms for Arabidopsis homologs of gene candidates and their interactors.**

**Table S10. Interactions between selected gene candidates and intermediate regulators.** Putative mechanisms of interactions are inferred based on available and accumulated evidence.
