# Supplementary material for: GWAS supported by computer vision identifies large numbers of candidate regulators of in planta regeneration in Populus trichocarpa
Source: G3 (Bethesda). 2024 Feb 7;14(4):jkae026. doi: 10.1093/g3journal/jkae026 (PMC10989874; doi:10.1093/g3journal/jkae026)
Supplement: jkae026_Supplementary_Data [file jkae026_supplementary_data.zip › Table_S9_G3-2023-404699.docx]

**Table S9. Abbreviations and acronyms for Arabidopsis homologs of gene candidates and their interactors**

| *Acronym/abbreviation* | *Full name* | *Other acronym/abbreviation* | *Other full name* |
| --- | --- | --- | --- |
| HLS1 | HOOKLESS 1 | COP3 | CONSTITUTIVE PHOTOMORPHOGENIC 3 |
| RSM3 | RADIALIS-LIKE SANT/MYB 3 | MEE3 | MATERNAL EFFECT EMBRYO ARREST 3 |
| EBF1 | EIN3-BINDING F BOX PROTEIN 1 |  |  |
| EIN3 | ETHYLENE-INSENSITIVE3 |  |  |
| MOS4 | MODIFIER OF SNC1,4 |  |  |
| MYC2 | MYELOCYTOMATOSIS-LIKE 2 | JAI1 | JASMONATE INSENSITIVE 1 |
| MPK3 | MITOGEN-ACTIVATED PROTEIN KINASE 3 |  |  |
| MPK6 | MITOGEN-ACTIVATED PROTEIN KINASE 6 |  |  |
| VQ (family) | VQ motif-containing protein family |  |  |
| SID1 | SALICYLIC ACID INDUCTION DEFICIENT 1 | EDS5 | ENHANCED DISEASE SUSCEPTIBILITY 5 |
| SNC1 | SUPPRESSOR OF NPR1-1, CONSTITUTIVE 1 |  |  |
| NPR1 | NONEXPRESSER OF PR GENES 1 | SAI1 | SALICYLIC ACID INSENSITIVE 1 |
| TGA6 | TGACG SEQUENCE-SPECIFIC BINDING PROTEIN 6 |  |  |
| NPR3 | NONEXPRESSER OF PR GENES 3 |  |  |
| NPR4 | NONEXPRESSER OF PR GENES 4 |  |  |
| JAZ (family) | JASMONATE-ZIM-DOMAIN PROTEIN family |  |  |
| TT8 | TRANSPARENT TESTA 8 |  |  |
| TT2 | TRANSPARENT TESTA 2 | MYB123 | MYB DOMAIN PROTEIN 123 |
| WD40 (family) | Transducin/WD40 repeat-like protein superfamily |  |  |
| COI1 | CORONATINE INSENSITIVE1 |  |  |
